# A Metastatic Intrahepatic Cholangiocarcinoma With HPCs Features: Report of a Case

**DOI:** 10.3389/fonc.2022.829235

**Published:** 2022-03-01

**Authors:** Qiang Fu, Pan Liu, Shangkun Jin, Xu Zhang, Chuanjiang Liu, Mingxing Hu, Yuzhu Wang, Hongwei Zhang, Tao Qin

**Affiliations:** ^1^ Department of Hepato-Biliary-Pancreatic Surgery, Henan Provincial People’s Hospital (People’s Hospital of Zhengzhou University), Zhengzhou, China; ^2^ Department of Hepato-Biliary-Pancreatic Surgery, People’s Hospital of Henan University, Zhengzhou, China

**Keywords:** intrahepatic cholangiocarcinoma, muc-ICC, mixed ICC, hepatic progenitor cells, gene mutation

## Abstract

Intrahepatic cholangiocarcinoma (ICC) is a highly lethal hepatobiliary neoplasm, which originates from the bile ducts proximal to the second-order division. ICC can be anatomically divided into two subtypes: the large duct type (mucin-production ICC, muc-ICC) and the small duct type (mixed-ICC) origins from hepatic progenitor cells (HPCs). The immunoreactivity of S100P and neural cell adhesion molecule (NCAM) are useful biomarkers to distinguish the two subtypes. In this study, we report a difficult-to-diagnose case of metastatic retroperitoneal tumor of occult hepatolithiasis-associated ICC. Besides, this case was both positive for S100P and NCAM, considered as a rare muc-ICC with the HPCs features. Tumor whole exome sequencing detection results by Genetron (China) revealed that there were 41 gene mutations in this patient. The SMAD4-p.His530ThrfsTer47 and KRAS-p.Gly12Val mutation might promote the occurrence and distant metastasis of the tumor.

## Introduction

Intrahepatic cholangiocarcinoma (ICC) has an increasing incidence worldwide and is the second most common primary hepatic malignancy after hepatocellular carcinoma. Cirrhosis, viral hepatitis, obesity-associated liver disease, diabetes, and the diseases leading to hepatobiliary fibrosis, such as primary sclerosing cholangitis, Caroli’s disease, hepatolithiasis, and liver fluke infractions, are risk factors for ICC. However, only 50% of ICC patients have identifiable risk factors (1). Surgery is still the only curative treatment for ICC, but only a minority of patients are suitable for surgery, and the current 5-year survival rate is to be only 25%–40% after surgery (2). The main reason for the low surgical resection rate is that ICC cannot be diagnosed in the early stage because ICC patients do not have specific early symptoms. Therefore, most ICC patients are already at the advanced stage when they go to the hospital (3). ICC patients usually present with intrahepatic mass lesions and symptoms such as abdominal pain, weight loss, and jaundice (4). Parts of ICC patients are misdiagnosed as carcinoma of unknown primary, such as hepatocellular carcinoma, lymphoma, and carcinoma of other types. Here, we present a rare case of metastatic ICC with specific pathological findings that is difficult to diagnose.

## Case Presentation

A 63-year-old woman was admitted to our hospital for having intermittent and severe mid-upper abdominal pain, along with the symptoms of back pain, anorexia, and weight loss of 15 kg for 1 month. This patient denied the history of cancer, surgery, smoking, alcohol drinking, illicit drug use, and blood transfusion. There was no similar patient in her family. Her BMI = 17.72 kg/m^2^. On physical exam, she was of middle nutrition and had no jaundice. Her abdomen was soft, while an approximately 6 cm hard mass without clear boundaries in mid-upper abdomen was found. The laboratory data revealed that the patient had normal routine blood test, hepatic function, renal function, and coagulation function. The serum levels of carbohydrate antigen 19-9 (CA19-9) (8.01 U/ml), cancer embryonic antigen (CEA) (2.24 ng/ml), CA-125 (19.71 U/ml), CA-242 (1.24 U/ml), alpha-fetoprotein (AFP) (1.39 ng/ml) were all within the normal range. Intraoperative ultrasonography did not detect fatty liver. The abdominal contrast-enhanced computed tomography (CT) scan and CT angiography showed a huge irregular neoplasm located at the retroperitoneum with edge intensifying (**Supplementary Figure 1A**). Furthermore, the left renal vessel and ureter were invaded combined with left uronephrosis (**Supplementary Figures 1B, E**). Besides, there was atrophy of the left hepatic lobe, and choledocholithiasis was accompanied by intra- and extrahepatic bile duct dilatation (**Supplementary Figures 1C, D**). The patient underwent the surgery of retroperitoneal tumor resection, left nephrectomy, splenectomy, left hepatic lobectomy, cholecystectomy, choledocholithotomy, and T tube drainage, which continued about 360 min, and 525 ml of blood was lost. No tumor mass was found in the left hepatic lobe under naked eye, and the retroperitoneal tumor was solid, not encapsulated, whitish in color, and had an irregular margin.

### HE Staining and Immunohistochemistry

All specimens were cut into 5-μm sections and stained with hematoxylin and eosin (HE) after fixation. Tissue sections were deparaffinized, subjected to antigen retrieval, and blocked with 5% bovine serum albumin (BSA) in phosphate-buffered saline (PBS) for 30 min. CA19-9, CK19, CK7, neural cell adhesion molecule (NCAM), S100P, CK20, AFP, and GATA-3 primary antibodies were purchased from Abcam Plc (Cambridge, UK). CEA, Muc-1, CK8/18, Ki67, P40, P53, c-kit, Mucin-2, SATB, and Villin primary antibodies were purchased from Cell Signaling Technology (Danvers, MA, USA) and Hep-par1primary antibody was purchased from Affinity Biosciences (Cincinnati, OH, USA). The sections were incubated with primary antibody overnight at 4°C, followed by the appropriate secondary antibody at room temperature for 1 h. All images were captured by confocal microscopy. Hematoxylin and eosin (HE) staining revealed that chronic inflammation of the intrahepatic bile duct and high-grade intraepithelial neoplasia of the glandular epithelium were commonly seen in the left hepatic lobe of 10 different layers (**Supplementary Figure 2A**). The retroperitoneal tumor was epithelial-derived malignancy with necrosis, considered poorly differentiated adenocarcinoma combined with extensive squamousness (**Supplementary Figure 2B**). Besides, scattered mucin-production adenocarcinoma areas (**Supplementary Figure 3A**) and hyperplastic ductular areas, characterized by ductular reaction-like anastomosing glands in fibrous stroma with mild atypia (**Supplementary Figure 3B**), were seen. Clear-cut hepatocellular differentiation was not seen. Tumor cells invaded the left renal hilum, ureter, and peripheral fibrous adipose tissue. Adenocarcinoma tissue is seen in perirenal lymph nodes (1/9), while Group 12/13/16 lymph nodes were negative (0/18). Immunohistochemistry of retroperitoneal tumor was positive for CA19-9, CEA, Muc-1, CK19, CK7, CK8/18, Ki67 (60%), P40, P53 (partial), c-kit, NCAM, and S100P (**Supplementary Figures 4A–L**) and negative for CK20, GATA-3, Mucin-2, SATB, Villin, AFP, and Hep-par1 (**Supplementary Figures 4M–S**).

### Tumor Whole Exome Sequencing Detection

After genomic DNA was extracted from the samples, the library was constructed by fragment and targeted capture of the target region. DNA sequencing was performed based on NovaSeq high-throughput sequencing platform. It was revealed that there were 41 gene mutations but no gene rearrangement and no gene copy number variation. The mutational genes included nonsense mutation (ACHE), frame shift mutation (ADAM7, ANXA5, C13orf45, KLRC4, SMAD4, USP32, and VPS13C), missense mutation (ADAMTS17, ADAMTS9, BCOR, CA12, CASZ1, CEACAM21, CECR6, DAB1, DHCR24, DICER1, FAM57B, HIST1H4J, HTR5A, IGDCC4, IRF2BP2, KLHL36, KRAS, LRRC8D, LSS, MEX3B, MGA, MICAL2, OLFML2B, PZP, REXO4, SEMA5B, SH3PXD2A, SH3TC2, SLC25A37, SYNE1, SYNGR3, and ZFHX4) and splicing mutation (KCND2) (**Supplementary Table 1**).

## Discussion

The immunohistochemical results of the retroperitoneal tumor of this case indicated that Muc-1, CK7, CK19, and CEA were positive, which suggested that the tumor might originate from the biliary tract system. Extensive sampling by the pathologist and surgical clinicians of the hole left hepatic lobe was proceeded, and an about 0.4 mm local invasion adenoma with malignant transformation was seen at last (**Supplementary Figure 2C**). After a multidisciplinary discussion of the pathologist, oncologist, and surgical clinicians, this case was diagnosed as a hepatolithiasis-associated intrahepatic cholangiocarcinoma (ICC) accompanied by retroperitoneal metastasis. Hepatolithiasis is a major risk factor of ICC (5). Approximately 5.3%–12.9% of hepatolithiasis cases are complicated with ICC (6).The incidence of hepatolithiasis-associated ICC is relatively insidious, and the clinical symptoms are often hepatolithiasis, biliary infection, or hepatatrophia, which are easy to cause clinically missed diagnosis. The surgical resection rate is low, and the overall prognosis is poor. It has been reported that the 5-year postoperative survival rate of hepatobiliary cholangiolithiasis combined with hepatobiliary carcinoma is only 3.0%–18.4% (7).

ICC can be anatomically divided into two subtypes, namely, the large duct type and the small duct type, which have different etiologies, molecular alteration, growth patterns, and clinical behaviors (8).The large duct type iCCA, which originate from large intrahepatic bile duct (such as segmental, area, and septal bile duct) lining of mucin-producing cylindrical cells, resembles extrahepatic cholangiocarcinoma. Thus, this subtype can also be called mucin-production ICC (muc-ICC). On the other hand, the small intrahepatic bile duct, such as interlobular bile duct and ductules, contains hepatic progenitor cells (HPCs), which have the ability of differentiating into hepatocytes and cholangiocytes and causing tumors during the differentiation process. HPCs-derived tumors (mixed-ICC) display varying hepatocytic and/or cholangiocytic differentiation characteristics within the same tumor (9). Even with the presence of diffuse dilation of the left intrahepatic bile duct, the local invasion adenoma in our case was initially considered to be located at the segmental or area bile duct. Because the primary tumor was too small for further immunohistochemical detection of markers of stem/HPCs, we could further analyze the characteristic and origin from the metastatic tumor. As shown in **Supplementary Figures 3A, B**, the immunohistochemistry of mucin-production adenocarcinoma areas was positive for S100P and NCAM, and the ductular areas were also positive for NCAM and S100P (partial). The immunoreactivity of S100P and NCAM represents a useful tool to distinguish mixed-ICC from muc-ICC. S100P expression is only seen in mucin-positive ICC areas in muc-ICC but not in the mixed-ICC, whereas NCAM is only immunoreactive in the hep-dif and ductular areas in mixed-ICC but not in muc-ICC (10, 11). This case was positive both for S100P and NCAM. Combining the local invasion adenoma with malignant transformation most probably located at the segmental or area bile duct, the patient was considered as a rarely particular subtype of muc-ICC with the HPCs features.

Among the mutation genes of this patient, the mutations of SMAD4 and KRAS were reported to be associated with the genesis and invasion of cholangiocarcinoma. The SMAD4-p.His530ThrfsTer47 mutation of this patient leads to the premature generation of a stop codon, which results in the impact of SMAD4. SMAD4 is the central molecule of the TGF-β signal pathway, which is related to the occurrence, development, and metastasis of cancer (12). Loss expression of SMAD4 promotes the transition from stone-containing intrahepatic bile ducts (IHD) to ICC. SMAD4 expression is related to the histological grade, clinical stage, and metastasis of ICC. The loss of SMAD4 expression in metastatic ICCs was significantly more severe compared with non-metastatic ICC (13). Besides, the KRAS-p.Gly12Val mutation (KRAS^G12D^) of this patient, in the conserved G box domain of KRAS protein, results in the activation of KRAS. KRAS^G12D^ activation leads to the development of invasive ICC with low penetrance and long latency. Latency was shortened by combining KRAS^G12D^ activation with heterozygous or homozygous deletion of p53, which also resulted in widespread local and distant metastasis (14). Until now, there are several drugs targeting KRAS in clinical trials for the treatment of cholangiocarcinoma, such as Trametinib, Binimetinib, and Selumetinib.

In conclusion, we here presented a rarely metastatic occult ICC with the stem/PSC features, which is valuable in terms of examining a rare phenomenon of ICC at the molecular level. It is a pity that we cannot assess the systemic metastases for the absence of PET-CT and just rely on the feedback provided by the patient to know the progress of the disease. The SMAD4-p.His530ThrfsTer47 and KRAS-p.Gly12Val mutation of this patient might promote the occurrence and distant metastasis of the tumor.

## Data Availability Statement

The original contributions presented in the study are included in the article/**Supplementary Material**. Further inquiries can be directed to the corresponding authors.

## Ethics Statement

Written informed consent was obtained from the individual(s) for the publication of any potentially identifiable images or data included in this article.

## Author Contributions

CL and SJ were involved in histopathological diagnosis. QF and PL wrote the first draft of the manuscript. TQ and MH were involved in surgery and tissue collection. YW and XZ performed laboratory experiment. TQ and HZ reviewed and edited the manuscript before submission. All authors contributed to the article and approved the submitted version.

## Funding

The prevalence study was funded by the National Natural Science Foundation of China (31671440).

## Conflict of Interest

The authors declare that the research was conducted in the absence of any commercial or financial relationships that could be construed as a potential conflict of interest.

## Publisher’s Note

All claims expressed in this article are solely those of the authors and do not necessarily represent those of their affiliated organizations, or those of the publisher, the editors and the reviewers. Any product that may be evaluated in this article, or claim that may be made by its manufacturer, is not guaranteed or endorsed by the publisher.
