# Supplementary material for: A Metastatic Intrahepatic Cholangiocarcinoma With HPCs Features: Report of a Case
Source: Front Oncol. 2022 Mar 1;12:829235. doi: 10.3389/fonc.2022.829235 (PMC8921981; doi:10.3389/fonc.2022.829235)
Supplement: Supplementary file 1 [file DataSheet_1.docx]

Supplementary Material

# Supplementary Figures and Tables

## Supplementary Figures Legends

FIGURE 1: Abdominal CT. (A, B, E) Contrast-enhanced CT and CT angiography showed a hugely irregular neoplasm located at the retroperitoneum with edge intensifying. Left renal vessel and ureter was invaded combined with left uronephrosis. (C-D) CT showed that the left hepatic lobe was atrophy and choledocholithiasis was seen in extrahepatic bile duct.

FIGURE 2: Histological features of HE staining. (A) Chronic inflammation and high-grade intraepithelial neoplasia of the glandular epithelium were common seen in left intrahepatic bile duct. (B) Epithelial-derived malignancy with necrosis, considered adenocarcinoma combined with extensive squamousness was seen in the retroperitoneal tumor. (C) Local invasion adenomawith malignant transformation, about 0.4mm, was seen in segmental or area bile duct.

FIURE 3: Histological features and immunohistochemistry of mucin-production adenocarcinama and ductular areas. (A) Mucin-production adenocarcinama areas were positive for S100P and NCAM. (B) Ductular areas were positive for NCAM and S100P (partial).

FIGURE 4: Immunohistochemistry of retroperitoneal tumor. (A-L) Immunohistochemical staining of CA19-9, CEA, Muc-1, CK19, CK7, CK8/18, Ki67 (60%), P40, P53 (partial), c-kit, NCAM, S100P were positive. (M-S) Immunohistochemical staining of negative for CK20, GATA-3, Mucin-2, SATB, Villin, AFP, Hep-par1.

## Supplementary Table

Table 1: the mutant genes of this patient

| Gene | [Mutation](javascript:;) [type](javascript:;) | Nucleotide change | Amino acid change |
| --- | --- | --- | --- |
| ACHE | nonsense mutation | c.450T>A | p.Tyr150Ter |
| ADAM7 | frame shift mutation | c.406_407dup | p.Asn136LysfsTer11 |
| ANXA5 | frame shift mutation | c.961dup | p.Ter321LeufsTer24 |
| C13orf45 | frame shift mutation | c.99_106del | p.Ser34IlefsTer36 |
| KLRC4 | frame shift mutation | c.268del | p.Thr90GlnfsTer2 |
| SMAD4 | frame shift mutation | c.1587dup | p.His530ThrfsTer47 |
| USP32 | frame shift mutation | c.2270del | p.Pro757HisfsTer2 |
| VPS13C | frame shift mutation | c.10115dup | p.Thr3373AspfsTer2 |
| ADAMTS17 | missense mutation | c.1303G>T | p.Asp435Tyr |
| ADAMTS9 | missense mutation | c.5695G>T | p.Val1899Phe |
| BCOR | missense mutation | c.4975G>A | p.Ala1659Thr |
| CA12 | missense mutation | c.409G>A | p.Gly137Arg |
| CASZ1 | missense mutation | c.631G>A | p.Ala211Thr |
| CEACAM21 | missense mutation | c.349G>A | p.Val117Ile |
| CECR6 | missense mutation | c.1434C>G | p.Asp478Glu |
| DAB1 | missense mutation | c.756G>A | p.Met252Ile |
| DHCR24 | missense mutation | c.1081C>T | p.Pro361Ser |
| DICER1 | missense mutation | c.1132A>G | p.Ile378Val |
| FAM57B | missense mutation | c.233C>T | p.Thr78Met |
| HIST1H4J | missense mutation | c.221C>T | p.Thr74Ile |
| HTR5A | missense mutation | c.412G>T | p.Asp138Tyr |
| IGDCC4 | missense mutation | c.503G>A | p.Gly168Glu |
| IRF2BP2 | missense mutation | c.1477C>T | p.Leu493Phe |
| KLHL36 | missense mutation | c.1535A>T | p.Asp512Val |
| KRAS | missense mutation | c.35G>T | p.Gly12Val |
| LRRC8D | missense mutation | c.260A>G | p.Asn87Ser |
| LSS | missense mutation | c.1673G>A | p.Arg558Gln |
| MEX3B | missense mutation | c.741T>A | p.Asp247Glu |
| MGA | missense mutation | c.6676C>T | p.Arg2226Trp |
| MICAL2 | missense mutation | c.1441C>T | p.Pro481Ser |
| OLFML2B | missense mutation | c.1960G>A | p.Ala654Thr |
| PZP | missense mutation | c.4112T>A | p.Leu1371Gln |
| REXO4 | missense mutation | c.787G>A | p.Ala263Thr |
| SEMA5B | missense mutation | c.1612C>T | p.Arg538Cys |
| SH3PXD2A | missense mutation | c.195G>T | p.Lys65Asn |
| SH3TC2 | missense mutation | c.2003C>A | p.Ala668Asp |
| SLC25A37 | missense mutation | c.401A>T | p.Asp134Val |
| SYNE1 | missense mutation | c.24344C>G | p.Thr8115Ser |
| SYNGR3 | missense mutation | c.398C>T | p.Thr133Met |
| ZFHX4 | missense mutation | c.7927G>A | p.Gly2643Arg |
| KCND2 | splicing mutation | c.1468-1G> A | N/A |
