# Supplementary material for: A Metastatic Intrahepatic Cholangiocarcinoma With HPCs Features: Report of a Case
Source: Front Oncol. 2022 Mar 1;12:829235. doi: 10.3389/fonc.2022.829235 (PMC8921981; doi:10.3389/fonc.2022.829235)
Supplement: Supplementary file 2 [file Presentation_1.pptx]

## Slide 1
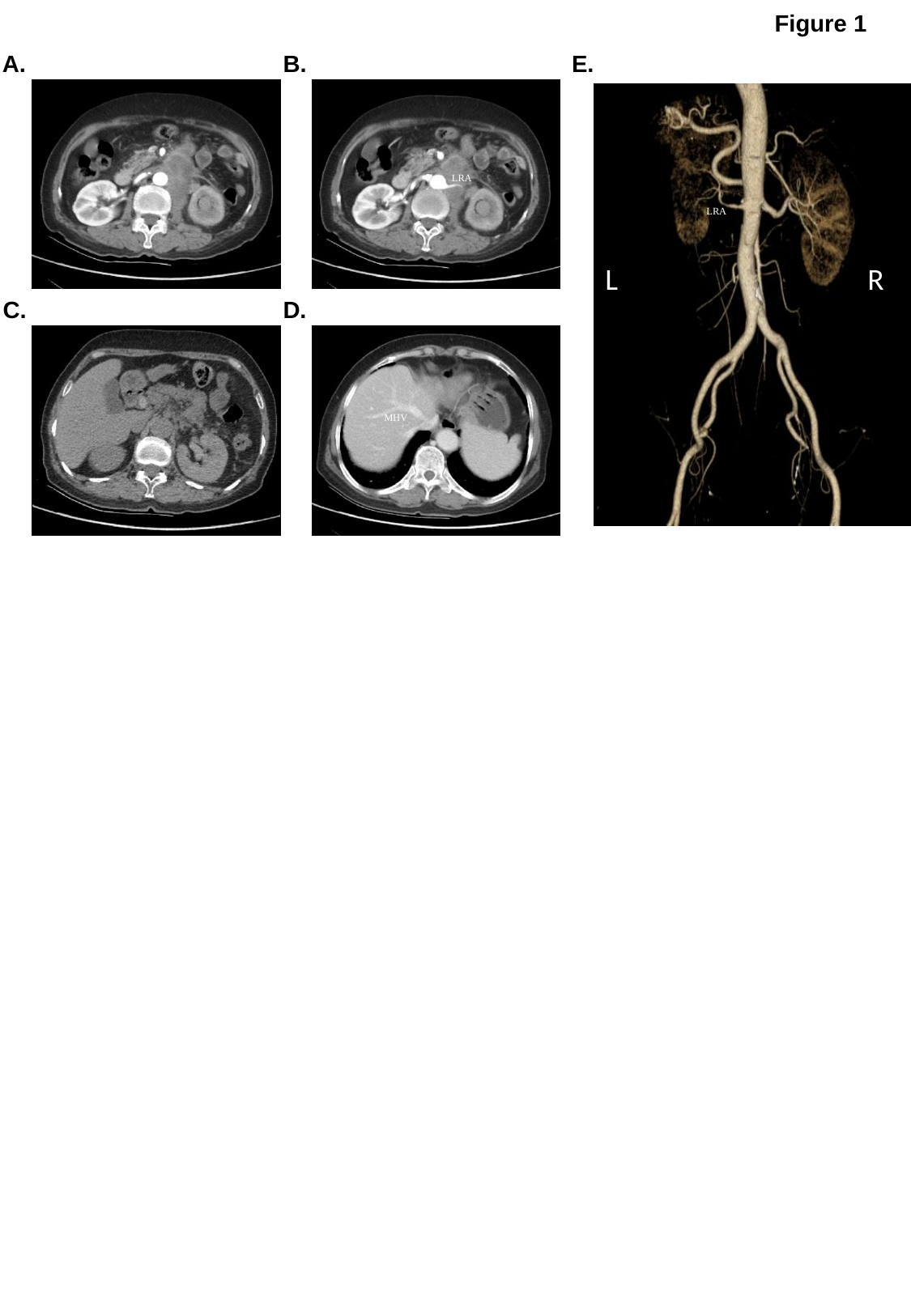

Figure 1
B.
A.
E.
LRA
LRA
L
R
LRA
C.
D.
MHV

## Slide 2
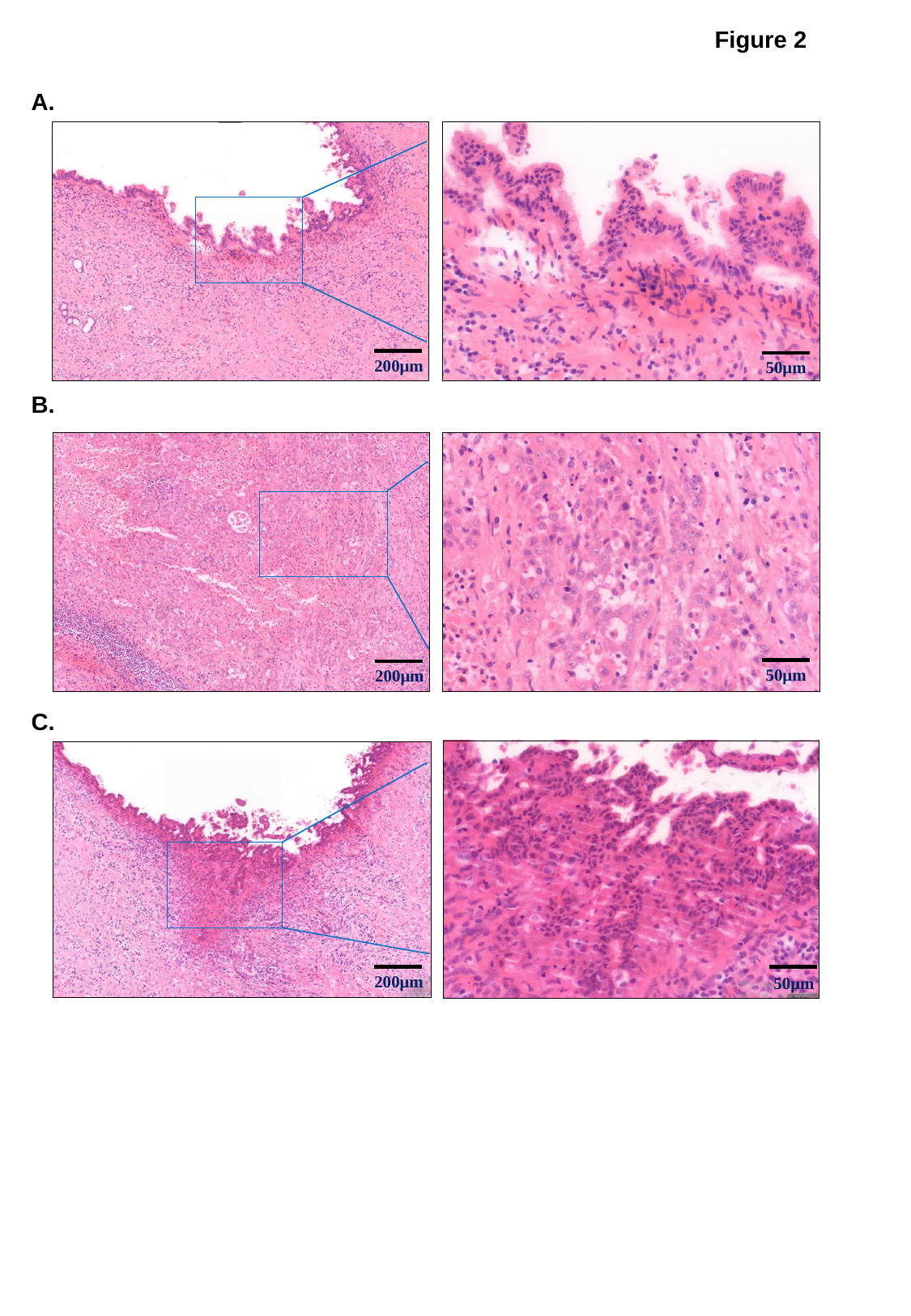

Figure 2
A.
200μm
50μm
50μm
50μm
B.
200μm
C.
200μm

## Slide 3
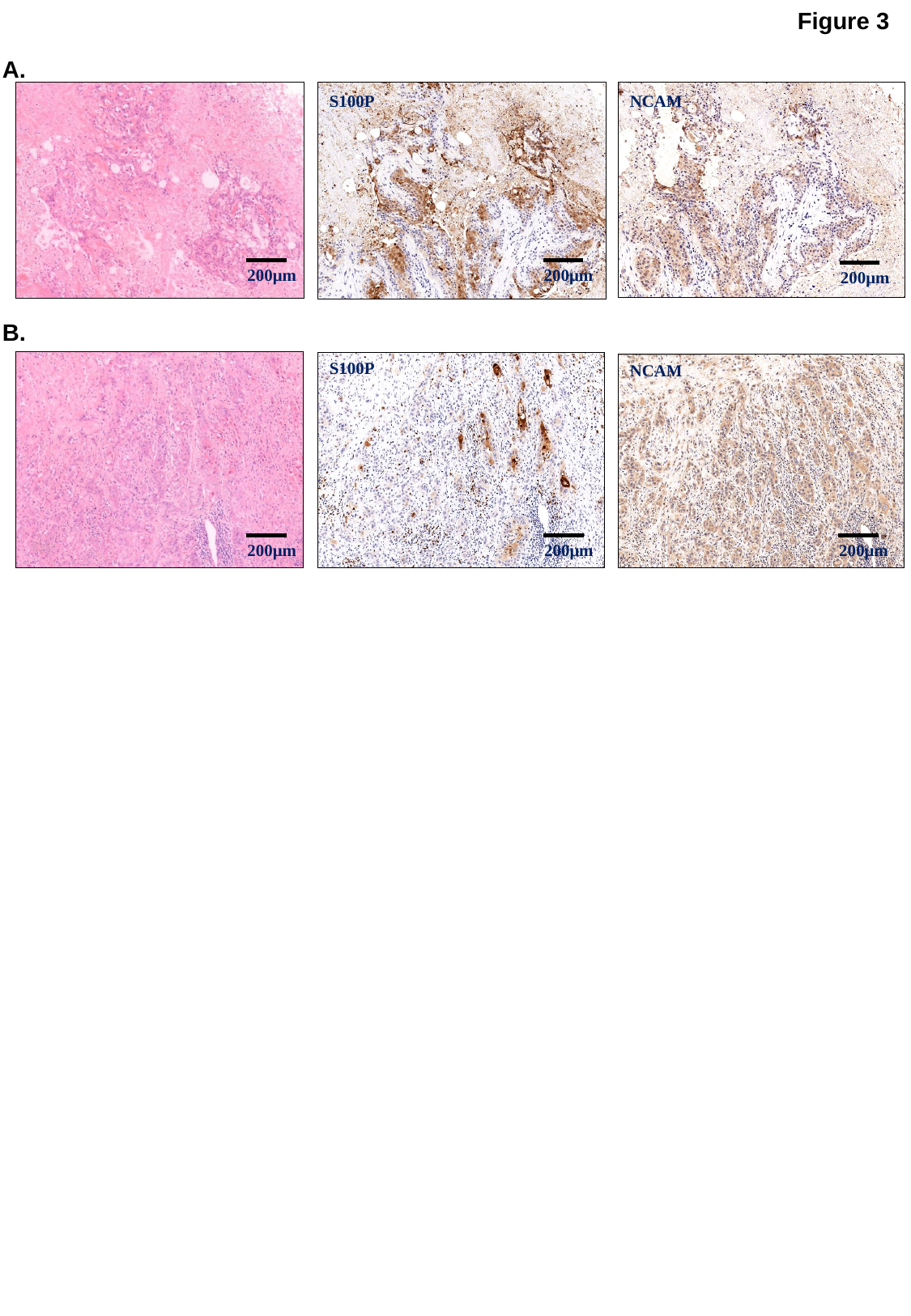

Figure 3
A.
S100P
NCAM
200μm
200μm
200μm
B.
S100P
NCAM
200μm
200μm
200μm

## Slide 4
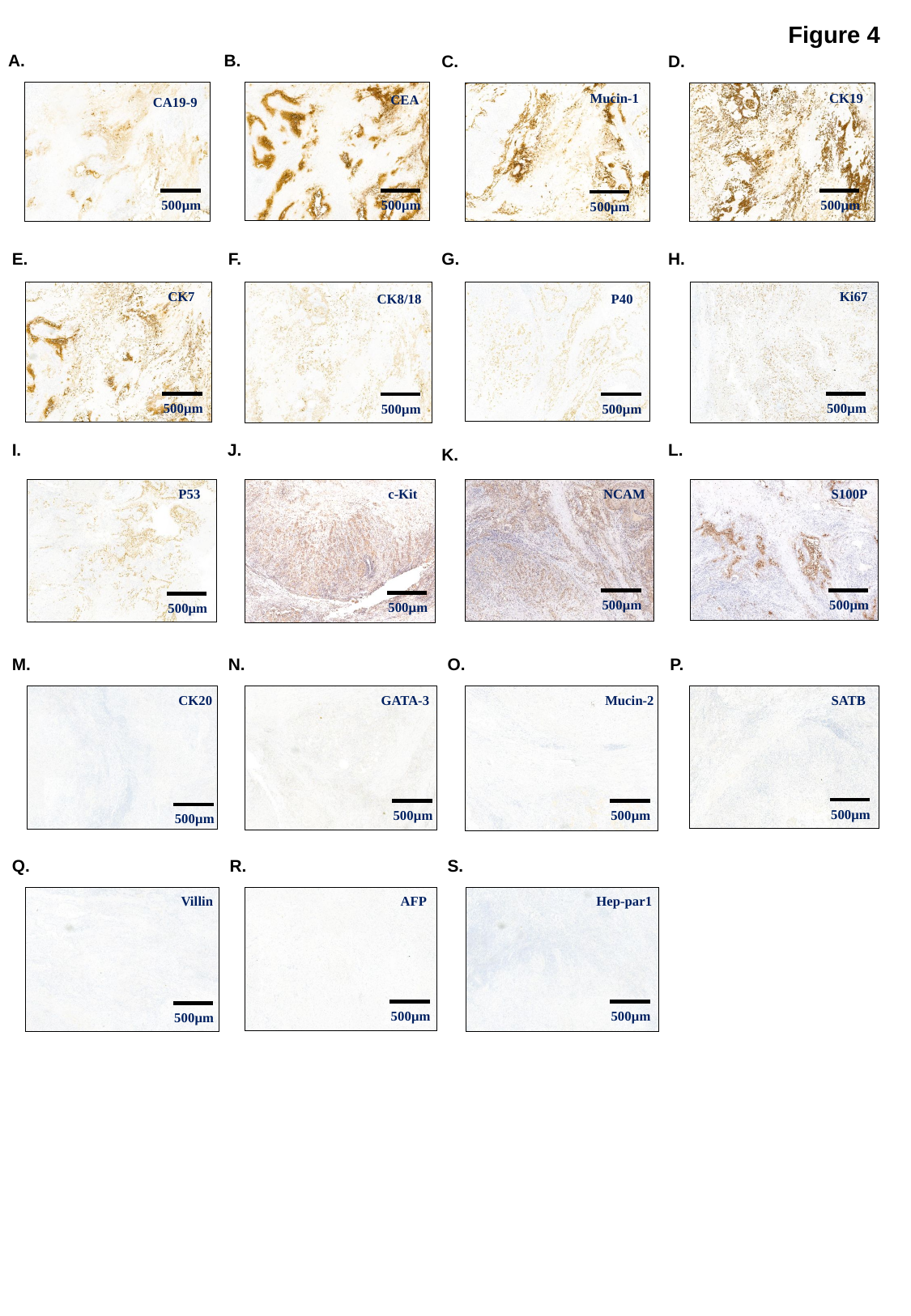

Figure 4
A.
B.
C.
D.
Mucin-1
CK19
CEA
CA19-9
500μm
500μm
500μm
500μm
E.
F.
G.
H.
CK7
Ki67
CK8/18
P40
500μm
500μm
500μm
500μm
I.
J.
L.
K.
P53
c-Kit
NCAM
S100P
500μm
500μm
500μm
500μm
M.
N.
O.
P.
CK20
GATA-3
Mucin-2
SATB
500μm
500μm
500μm
500μm
Q.
R.
S.
Villin
AFP
Hep-par1
500μm
500μm
500μm
